# Biparatopic anti-PCSK9 antibody enhances the LDL-uptake in HepG2 cells

**DOI:** 10.1038/s41598-024-66290-9

**Published:** 2024-07-03

**Authors:** Xinyang Li, Wei Zhang, Yu Shu, Rui Huo, Chengyang Zheng, Qi Qi, Pengfei Fu, Jie Sun, Yuhuan Wang, Yan Wang, Juxu Lu, Xiangjie Zhao, Guoyou Yin, Qingqing Wang, Jun Hong

**Affiliations:** 1 College of Life Science and Engineering, Henan University of Urban Construction, Ping Dingshan, 467036 China; 2 School of Public Health and Emergency Management, Southern University of Science and Technology, Shenzhen, 518055 Guangdong China; 3 Employment and Business Startup Service Center, Henan University of Urban Construction, Ping Dingshan, 467036 China

**Keywords:** Epitope, Heavy chain antibody, LDLR, LDL-c, Biochemistry, Drug discovery

## Abstract

Proprotein convertase subtilisin/kexin type 9 (PCSK9) has emerged as a promising therapeutic target to reduce lipids. In 2020, we reported a chimeric camelid-human heavy chain antibody VHH-B11-Fc targeting PCSK9. Recently, it was verified that VHH-B11 binds one linear epitope in the PCSK9 hinge region. To enhance its druggability, we have developed a novel biparatopic B11-H2-Fc Ab herein. Thereinto, surface plasmon resonance (SPR) confirmed the epitope differences in binding-PCSK9 among VHH-B11, VHH-H2 and the approved Repatha. Additionally, SPR revealed the B11-H2-Fc exhibits an avidity of approximately 0.036 nM for PCSK9, representing a considerable increase compared to VHH-B11-Fc (~ 0.69 nM). Moreover, we found the Repatha and B11-H2-Fc exhibited > 95% PCSK9 inhibition efficiency compared to approximately 48% for the VHH-Fc at 7.4 nM (*P* < 0.0005). Further, we verified its biological activity using the human hepatoma cells G2 model, where the B11-H2-Fc exhibited almost 100% efficiency in PCSK9 inhibition at only 0.75 μM. The immunoblotting results of low-density lipoprotein cholesterol (LDL-c) uptake assay also demonstrated the excellent performance of B11-H2-Fc on recovering the LDL-c receptor (LDLR), as strong as the Repatha (*P* > 0.05). These findings provide the first evidence of the efficacy of a novel Ab targeting PCSK9 in the field of lipid-lowering drugs.

## Introduction

Hyperlipidaemia, characterized by elevated levels of cholesterol, triglycerides, glycolipids, and phospholipids in the body, is a leading cause of vascular diseases such as atherosclerosis^1^. This condition poses a significant health risk, comparable to hypertension, and has been associated with the progression of various diseases, including liver cirrhosis, diabetes, and pancreatitis^2,3^. With the increasing uptake of high-fat diets, hyperlipidemia has become one of the main threats to people’s health. Current lipid-lowering drugs primarily rely on statins, which have shown clinical effectiveness. However, prolonged statin use can lead to drug intolerance and severe side effects such as abnormal liver function and rhabdomyolysis^4,5^. Therefore, it is necessary to identify other biological targets to reduce blood lipids.

An safe and efective biotarget is the ninth member of the *protein convertase subtilisin/kexin* family (*PCSK9*). As early as 2003, it was reported by a human genetics studies^6^. In addition to the *LDLR* and *apolipoprotein B* genes, the researchers found that the *PCSK9* gene is the third locus of autosomal dominant hypercholesterolemia^7^. LDL-c plays a vital role in cholesterol metabolism by binding to the LDLR. However, high level PCSK9 also binds LDLR, which would cause the abnormal elevation of plasma LDL-c and the abnormal degradation of LDLR^8^. Therefore, PCSK9 Ab inhibitors have become a desirable alternative in addition to statins. To date, three lipid-lowering Abs including Repatha against PCSK9 have been approved worldwide^9,10^, with several others in clinical trials or under consideration for market approval in China and abroad^11^. Despite their efficacy, these conventional human Abs have limitations. Firstly, the majority of them only bind to a single epitope within the catalytic region of PCSK9, resulting in a limited lipid-lowering efficiency of approximately 50%. Secondly, they are expensive, with the Repatha and Praluent costing up to $5850 per year^12^. Because the Repatha is more representative than the others, we have used it as the positive control in this study.

With the continuous development of Ab drugs, in order to address the unmet needs for clinical disease treatment, Ab drugs are evolving towards miniaturization, bifunctionality, bispecificity or conjugation with small molecule drugs. Among these advancements, the easily modifiable single-domain antibody (sdAb) and HcAb are emerging as a promising avenue for future Ab drug development^13^. Several sdAb/HcAb drugs such as Cablivi (VHH-VHH expressed by yeast), envafolimab and KN046 (VHH-Fc) have already been approved for clinical use^14–18^, highlighting their high druggability.

Previously, we reported the development of a camelid-human chimeric HcAb, VHH-B11-Fc, developed based on the llama Ab library using phage display technology^19,20^. This Ab exhibited 100% inhibition of PCSK9 in hepatocellular carcinoma models at a specific concentration (1.5 µM). Moreover, we characterized the interaction epitopes of VHH-B11 in the hinge region of PCSK9 as described previously^19^. In this study, we aimed to enhance the druggability of VHH-B11-Fc in lipid-lowering by designing a novel antibody^20–23^.

## Results

### Phage display and sdAb affinity test

**Figure 1:**
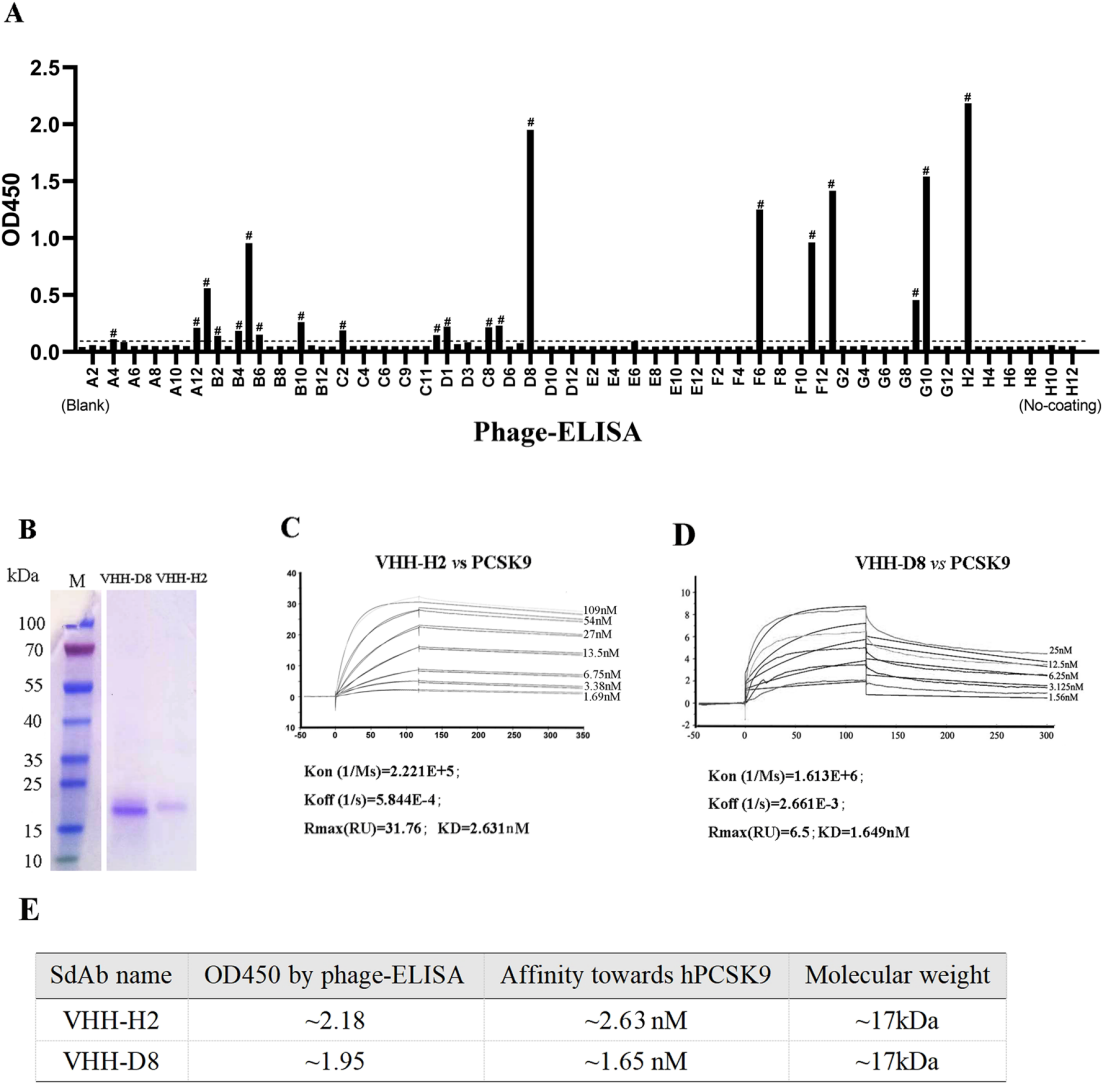
Phage-ELISA assay, SDS-PAGE and affinity tests of two novel sdAbs. (**A**) The phage-ELISA results. The horizontal axis represents different samples in 96 well plate. The vertical axis represents the OD450 value. ‘(Blank)’ refers to the PBS control (A1–A3 wells) of the ELISA assay. ‘(No coating)’ refers to the no-PCSK9 coating negative control (H10–H12 wells) of the ELISA assay. The star (#) represents positive (the OD450 ratio of the experimental group/blank group ≥ 2.1). (**B**) LaneM: the protein marker (Fermentas, USA). Left lane: the VHH-D8 antibody (~ 17 kDa) expressed by HB2151 *E. coli* (*pMECS-D8*); Right lane: the VHH-H2 antibody (~ 17 kDa) expressed by HB2151 *E. coli* (*pMECS-H2*); (**C**) Affinity determination between VHH-H2 and human PCSK9-his. (**D**) Affinity determination between VHH-D8 and human PCSK9-his. The horizontal axis represents the timeline. The vertical axis represents the relative response unit. (**E**) A list of the main properties of the screened sdAbs (VHH-H2 and VHH-D8).

We employed phage display to identify sdAbs with high affinities for PCSK9. After four rounds of phage rescue and specific panning, we acquired twenty sdAbs with relatively high ELISA responses (marked with “#”, Fig. 1A). After Sanger sequencing and excluding clones with having the same sequences, we selected two sdAbs with considerably high ELISA binding responses, VHH-H2 (OD450 ≈ 2.18) and VHH-D8 (OD450 ≈ 1.95) (Fig. 1A, Table S1). Subsequently, the expression of these his-tagged sdAbs were induced in HB2151 *Escherichia coli* (*E. coli*) cells (*pMECS-VHH*) using isopropyl β-D-thiogalactoside (IPTG) induction, followed by Ni-chelating affinity purification. The SDS-PAGE analyses confirmed the expected molecular weight of approximately 17 kDa for both sdAbs (Fig. 1B, Fig. S1). The affinities of VHH-H2 and VHH-D8 toward hPCSK9 were further determined using surface plasmon resonance (SPR) yielding values of ~ 2.63 nM and ~ 1.65 nM, respectively (Fig. 1C/D). However, VHH-D8 exhibited a faster dissociation rate based on the kinetic curves. Therefore, VHH-H2 was selected as the preferred sdAb for further investigations (Fig. 1E).

### Epitope binning by SPR

**Figure 2:**
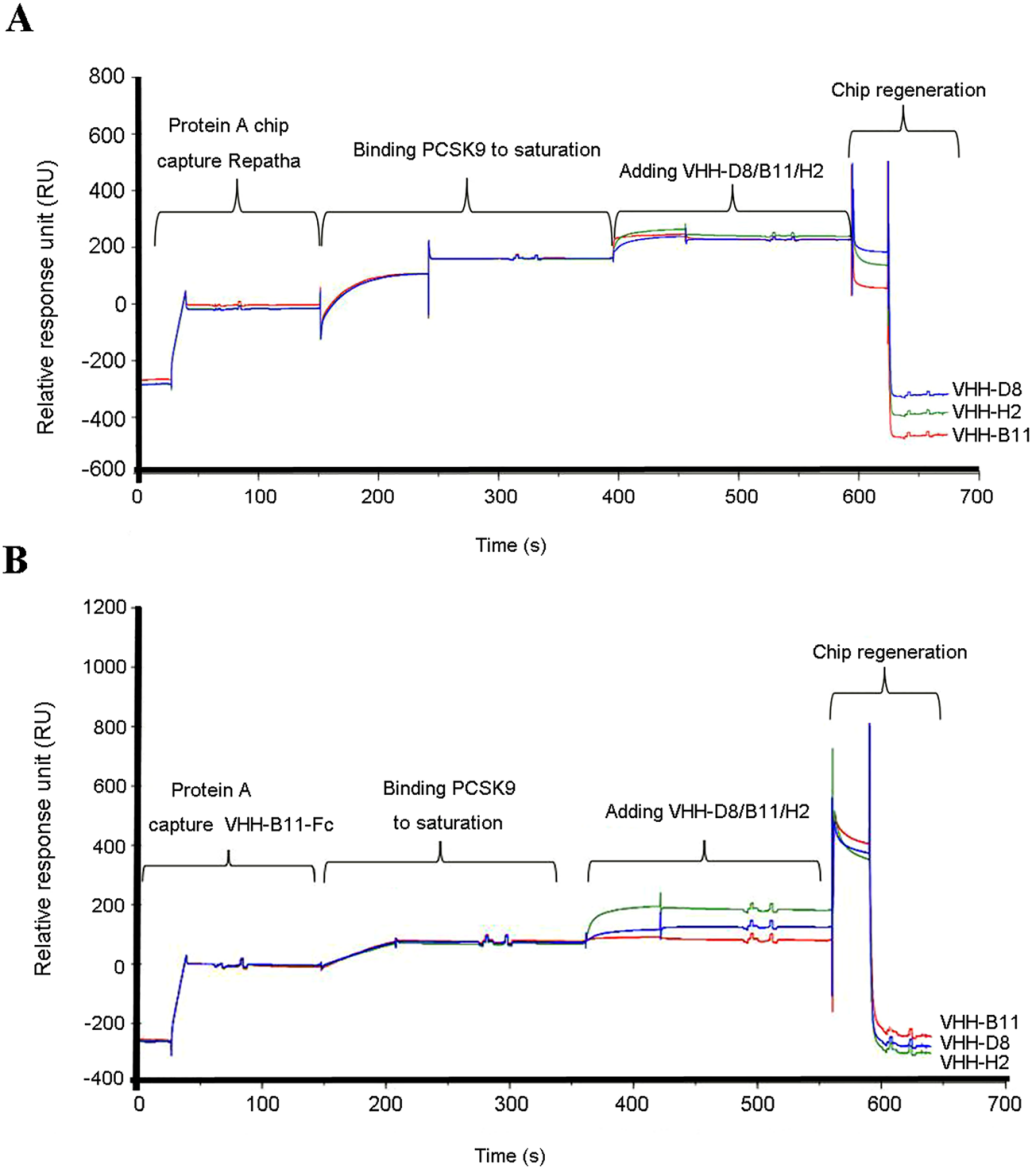
The epitope binning assay- comparison between VHH-H2/D8/B11 and Repatha by SPR. The epitope binning assay consists of four steps: chip capturing; binding PCSK9 to saturation; adding secondary antibody; Protein A chip regeneration. (**A**) It shows the binding-epitope differences between VHH-H2/D8/B11 and Repatha by SPR. (**B**) It shows the binding-epitope differences between VHH-H2/D8 and VHH-B11 by SPR. The horizontal axis represents the timeline. The vertical axis represents the relative response unit.

We then evaluated the epitope differences in binding-PCSK9 among Repatha, VHH-B11, VHH-H2, and VHH-D8. First, the Repatha was captured using a Protein A chip (0–150th sec), and its binding epitope on the catalytic region of hPCSK9 was saturated (150–400th sec). We normalized the response unit (RU) value after saturation (vertical coordinate), with 0 RU assigned at ~ 50th sec. (Fig. 2A). Starting from the 400th sec, when injecting three VHHs sequentially, the red curve exhibited a pronounced upward increase, confirming that VHH-B11 differed from Repatha in terms of PCSK9-binding epitopes. Similarly, VHH-H2 (green curve) and VHH-D8 (blue curve) also increased, indicating that they also differed from Repatha in terms of PCSK9-binding epitopes (400–580th sec). This is followed by chip regeneration.

Subsequently, the VHH-B11-Fc was captured on the Protein A chip (0–150th sec), followed by saturation (150–360th sec) of its binding epitopes by injecting the hPCSK9-his protein starting from the 150th sec (Fig. 2B). When resampling the 6his-tagged VHH-B11 (at the 360th sec, Fig. 2B), the red curve exhibited a minimal increase, indicating the feasibility of this method. We then separately loaded VHH-H2 (green curve) and D8 (blue curve), both showing a rise at the 360th sec. However, the green curve representing VHH-H2 displayed a more pronounced rise, whereas the blue curve of VHH-D8 seemed to have a limited rise (360–560th sec) followed by chip regeneration.

These findings demonstrate the epitope differences in PCSK9 binding among Repatha, VHH-B11, and VHH-H2.

### Design and construction of the biparatopic antibody and avidity tests

**Figure 3:**
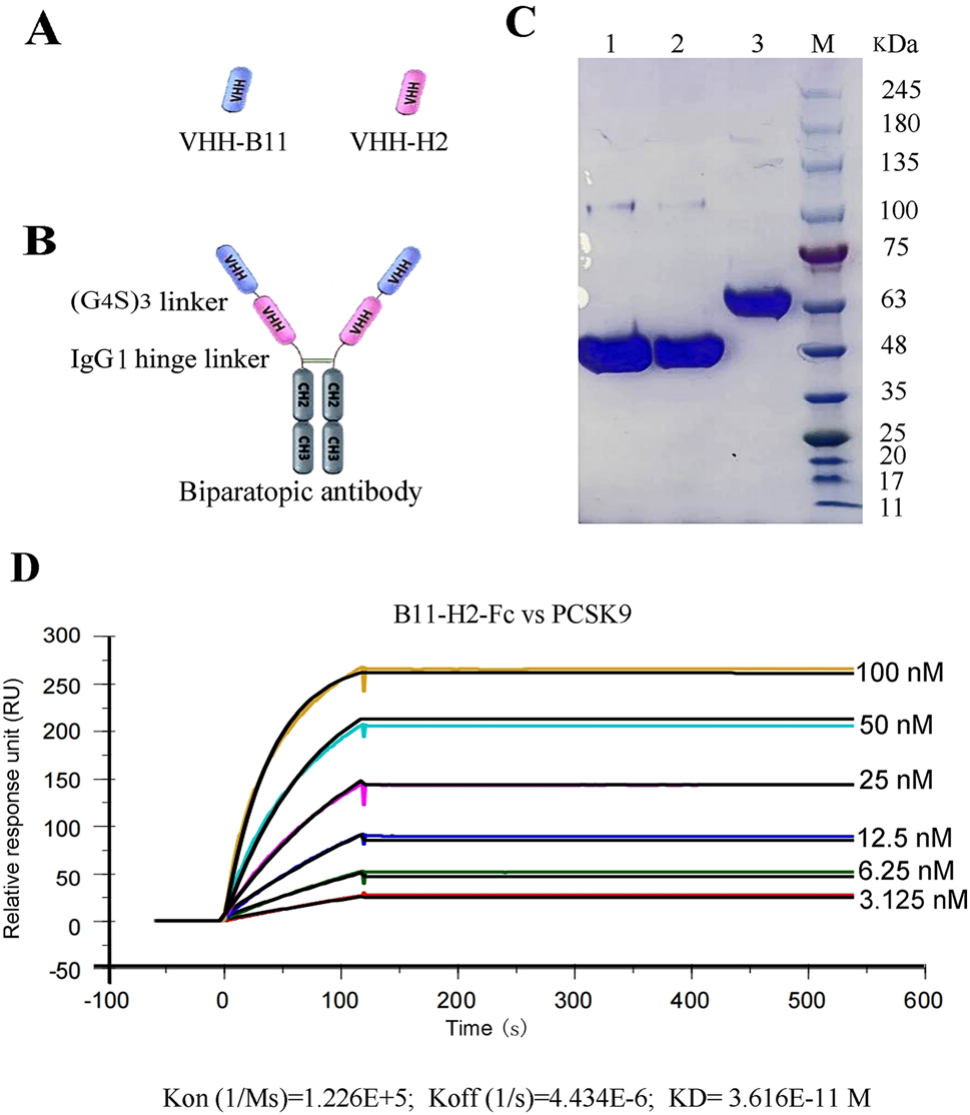
Schematic representation, SDS-PAGE and SPR tests of B11-H2-Fc. (**A**, **B**) Schematic representation of two VHHs and biparatopic B11-H2-Fc configurations. “(G_4_S)_3_” is a 15- amino acid peptide linker (GGGGSGGGGSGGGGS). “IgG1-hinge linker” means the hinge region between the human IgG1 Fab and Fc. (**C**) Lane M: the protein marker (Fermentas, USA). Lane 1: the VHH-B11-Fc antibody (~ 45 kDa) expressed by HEK293F (*pFUSE-hIgG1-B11*); Lane 2: the VHH-H2-Fc antibody (~ 45 kDa) expressed by HEK293F (*pFUSE-hIgG1-H2*); Lane 3: the B11-H2-Fc antibody (~ 65 kDa) expressed by HEK293F (*pFUSE-hIgG1-B11-H2*) under reducing conditions. (**D**) SPR tests between B11-H2-Fc and human PCSK9-his. The horizontal axis represents the timeline. The vertical axis represents the relative response unit.

Based on the above findings, to enhance the efficiency of B11-Fc in inhibiting PCSK9, we designed a novel biparatopic Ab, VHH-B11-VHH-H2-Fc (B11-H2-Fc), by tandemly linking VHH-B11 and VHH-H2 and then fusing them with human IgG1 Fc. The structure of the Ab is shown in Fig. 3A,B. Moreover, Fig. 3C and Fig. S2 show a gel plot of the Ab after expression in HEK293F and purification by a Protein A column (under reducing conditions), indicating molecular weights of 45 kDa (VHH-Fc Abs) and 65 kDa (VHH-VHH-Fc Ab) under reducing conditions, as expected.

In addition, we used SPR to determine the avidity of the Ab for hPCSK9, resulting in an avidity value of approximately 0.036 nM (Fig. 3D), which represents a considerable improvement compared to B11-Fc antibody (~ 0.69 nM)^19,20^.

### ELISA assay

**Figure 4:**
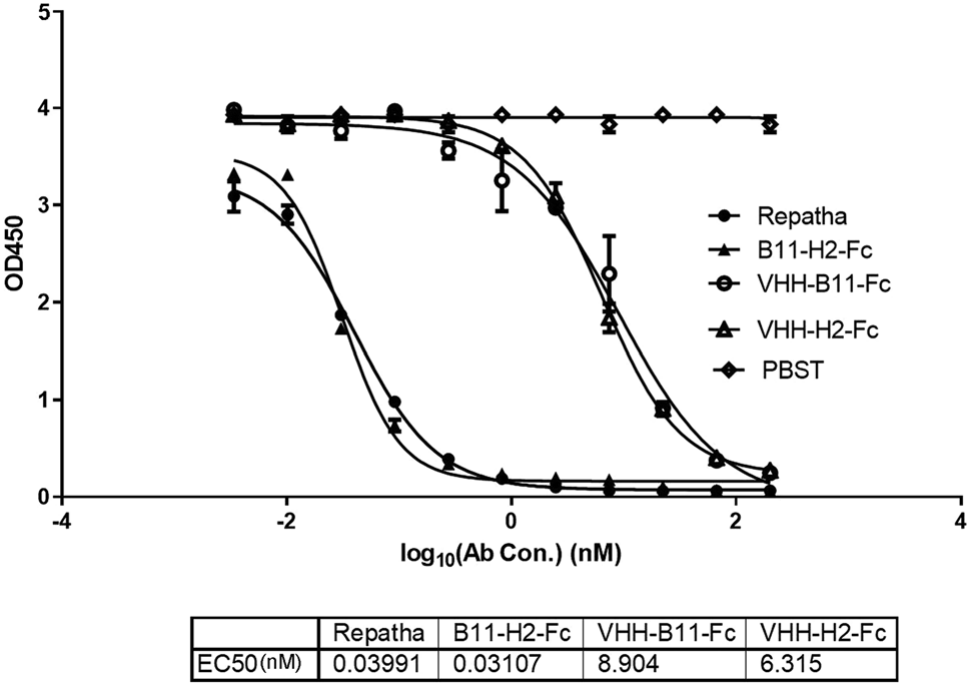
ELISA assay. The ELISA assay was performed to verify the efficiency of the novel biparatopic antibody in inhibiting PCSK9’s binding to LDLR. The serially diluted antibodies—the Repatha, VHH-B11-Fc, VHH-H2-Fc or B11-H2-Fc were incubated with PCSK9-hFc to inhibit the binding to LDLR. The EC50 value is listed below for comparing the inhibition ability between the antibodies. The horizontal coordinate is the value after taking log10 of the antibody concentration. The vertical axis represents the OD450 value (optical density value at 450 nm) by microplate reader.

To further confirm the potential of this biparatopic Ab to inhibit PCSK9, we performed ELISA. The purpose was to compare the efficacy of inhibiting PCSK9 between these Abs at the protein level. With increasing Ab concentrations, all Abs showed progressively greater ability to inhibit PCSK9 binding to LDLR (Fig. 4). This suggests weakened the OD450 signals of the LDLR-PCSK9 mixture with increasing Ab concentrations. However, all Abs eventually exhibited satisfactory PCSK9 inhibition (> 93%) at 200 nM (Fig. S3). Our results indicated that the Repatha and biparatopic B11-H2-Fc Ab have a similar EC50 value (~ 0.036 nM). Additionally, the average EC50 value of the VHH-Fc antibody was approximately 7.61 nM.

Furthermore, Fig. S3 presents the inhibition rates more graphically in the form of a bar chart. We found that the Repatha and biparatopic B11-H2-Fc Ab exhibited > 95% PCSK9 inhibition efficiency compared to approximately 48% for the VHH-Fc Ab at 7.4 nM (*P* < 0.0005). Notably, when the novel Ab was added at 200 nM, the inhibition rate reached nearly 100%, whereas that of VHH-Fc Abs remained approximately 93%. The ELISA results revealed comparable performances between VHH-B11-Fc and VHH-H2-Fc. Moreover, we found that the efficacy of this biparatopic Ab exhibited comparable performance with the approved Repatha (*P* > 0.05). These findings collectively highlight the strong potential of the biparatopic Ab B11-H2-Fc, in effectively inhibiting PCSK9 at the protein level.

### LDL-uptake assay in HepG2 cells

**Figure 5:**
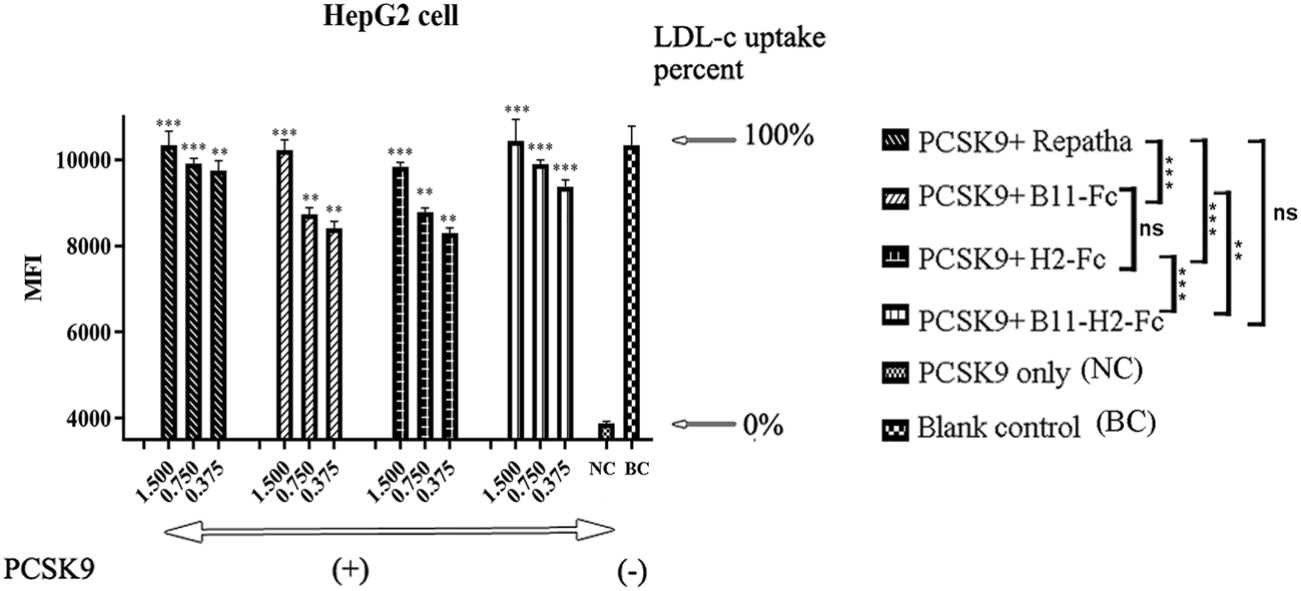
LDL-uptake assay. The LDL-uptake test in HepG2 cell models. The horizontal axis represents six different groups. The vertical axis represents mean fluorescence intensity (MFI) of the intracellular LDL-BODIPY, which reflects the levels of the LDL-c metabolism. The right two groups were respectively served as the blank control (BC, no addition of PCSK9 and its inhibitor) and negative control (NC, only addition of 0.1 μM PCSK9). The left four columns represent the groups of the Repatha, VHH-B11-Fc, VHH-H2-Fc and B11-H12-Fc respectively in three doses (0.375, 0.750 and 1.500 μM). LDL-c uptake percent was given on the right (←). “(+)” and “(−)” refers to the addition and absence of hPCSK9 or Abs, respectively. The data were expressed as mean ± SD. Statistical significance was determined using Student’s paired *t*-test. *P* < 0.05 was considered as statistically significant, compared with the negative control group (*P < 0.05, **P < 0.005, ***P < 0.0005, ns: not significant).

It is known that LDL binds to LDLR on the cell surface, facilitating cholesterol transport through receptor-mediated endocytosis^24^. However, the presence of abnormally high levels of PCSK9 considerably inhibits this binding process^24^. A labeled LDL complex (LDL-BODIPY) was used to assess the inhibitory effects of the Abs on PCSK9, and the mean fluorescence intensity (MFI) was measured as an indicator of LDL-c endocytosis. Theoretically, a higher fluorescent signal indicates stronger inhibitory activity of the Abs. As presented in Fig. 5, we found that the addition of PCSK9 alone resulted in a 60% reduction in fluorescence in human hepatocytes (HepG2), consistent with previous reports^9,10^. Furthermore, upon the addition of 1.5 µM Abs, except for VHH-H2-Fc, the PCSK9-inhibiting rates of all other Abs reached nearly 100% (Fig. 5). Almost all Abs exhibited a concentration-dependent inhibition pattern against PCSK9. However, at a concentration of 0.75 µM, the two VHH-Fc Abs exhibited a significantly lower inhibitory performance than the B11-H2-Fc Ab or Repatha in HepG2 cells (*P* < 0.005). The addition of 0.375 µM pronounced this downward trend further for all Abs. Nevertheless, 0.375 µM B11-H2-Fc inhibited approximately 80% hPCSK9 antigen, whereas the same concentration of Repatha inhibited approximately 85% hPCSK9. Therefore it was recognized that the biological activity of B11-H2-Fc in inhibiting PCSK9 was as strong as that of the approved Repatha at the cellular level (*P* > 0.05).

### Western blotting analyses of LDLR

**Figure 6:**
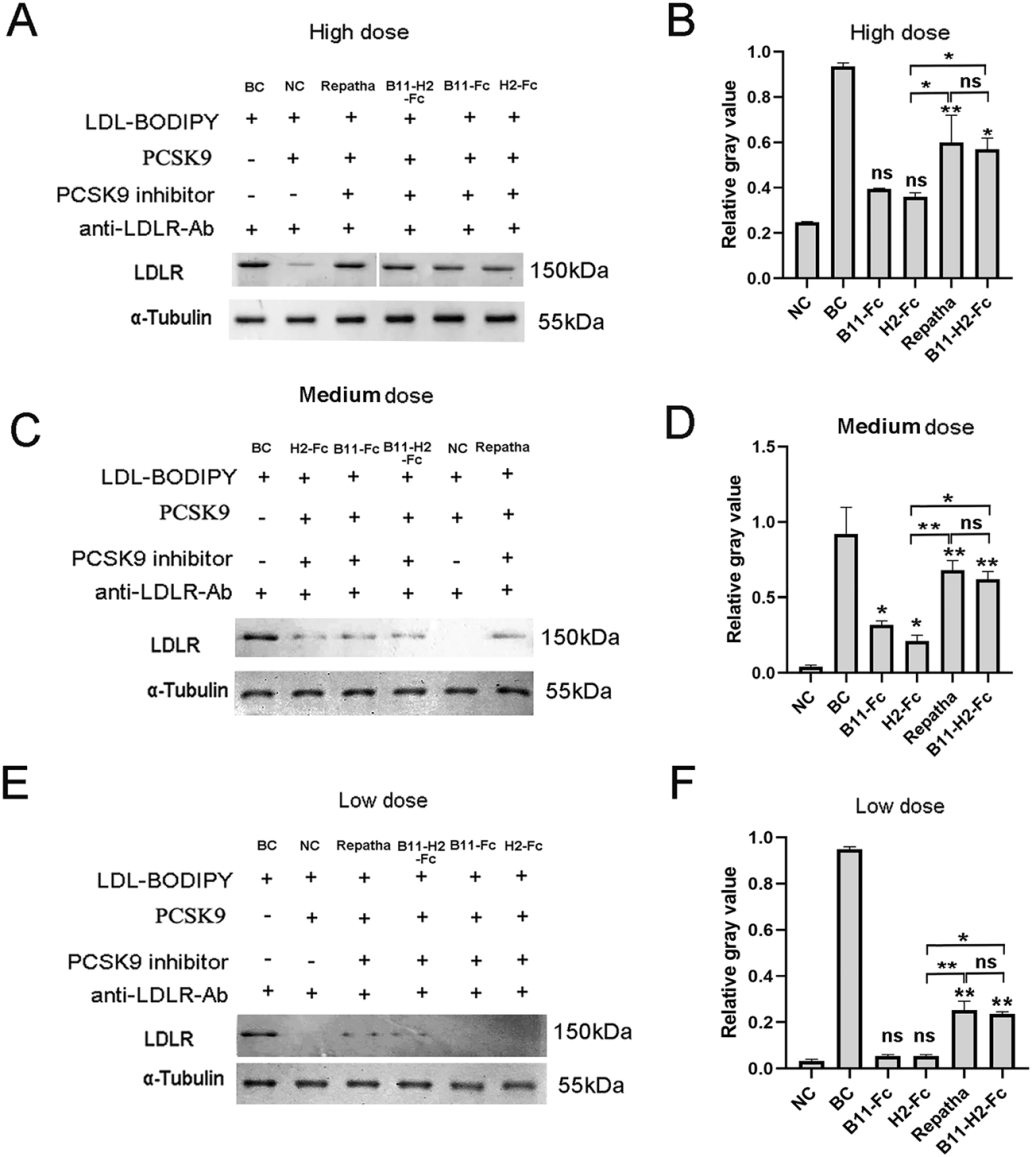
Western blotting and quantitative analyses. The western blotting and quantitative analyses were performed to determine the expression level of LDLR (~ 150 kDa) of hepatocytes. The α-tubulin (~ 55 kDa) was served as the internal control protein. Because the LDLR and α-tubulin are incubated with different Abs, we did not perform co-incubation to avoid non-specific bands. Under the same conditions, each sample was divided equally into two equal parts. Then they were loaded to two same SDS-PAGE gels, one for the test of LDLR and the other for α-tubulin. The full-length blots were shown in Fig. S4A-D. The results are presented in the form of high (1.5 μM) (**A**), medium (0.75 μM) (**C**) and low (0.375 μM) (**E**) doses. BC: the blank control group, referring to no addition of PCSK9 and its inhibitor; NC: the negative control, referring to only addition of 0.1 μM PCSK9. “+” and “−” refers to the addition and absence of the reagents such as LDL-BODIPY, PCSK9 or its Abs in above LDL-uptake assays. And “anti-LDLR-Ab, +” refers to the addition in the western blotting assays. The quantitative analyses of high (1.5 μM) (**B**), medium (0.75 μM) (**D**) and low (0.375 μM) (**F**) doses were performed by software “ImageJ”. The blotting results shown are representative of at least two independent experiments. Normalization was processed by dividing the greyscale value of the experimental group by that of the respective α-tubulin protein. Statistical significance was determined using Student’ s paired *t*-test. *P* < 0.05 was considered as statistically significant, compared with the negative control group (*P < 0.05, **P < 0.005, ***P < 0.0005, ns: not significant).

We further performed western blotting (WB) analyses to evaluate the expression levels of LDLR of hepatocytes in the LDL-uptake assay. The immunoblotting results of three dose groups are shown in Fig. 6 and Fig. S4. Theoretically, the bands of the BC (no addition of PCSK9 and its inhibitor) groups should exhibit the highest intensity of the LDLR, because the cell surface LDLR of the BC group was not endocytosed and degraded. In contrast, in the NC group (only the addition of the PCSK9), part of the surface LDLR and PCSK9 formation complex was endocytosed and degraded. As a result, the band intensity of NC groups should be the lowest. Under high dose (1.5 µM) Ab-adding conditions, all four kinds of Ab groups exhibited approximately 150 kDa LDLR bands on the membrane (Fig. 6A). Quantitative analyses show that the B11-H2-Fc group has the similar grayscale levels with that of Repatha (Fig. 6B), consistent with the findings in Fig. 5. Subsequently, under medium-dose PCSK9-inhibiting Ab conditions (0.75 µM), distinct differences in the grayscale intensity of bands between each group became apparent. The bands in the B11-H2-Fc group are not only as bright as that in the Repatha group (*P* > 0.05), but they are significantly brighter than those in the two VHH-Fc Abs (*P* < 0.05, Fig. 6C/D). The results for the low dose (0.375 µM) conditions can be referred to Fig. 6E/F. Similarly, the band of the B11-H2-Fc or Repatha group appeared significantly brighter than those of two VHH-Fc Abs (*P* < 0.05), consistent with the findings in Fig. 5. The brightness of the bands in the B11-H2-Fc group was similar to that of the Repatha group (*P* > 0.05). α-Tubulin (55 kDa), a housekeeping protein, was used as the internal control. Again, it is demonstrated that the biological activity of B11-H2-Fc in inhibiting PCSK9 is as strong as that of the approved Repatha at the cellular level (*P* > 0.05).

## Discussion

During PCSK9 Ab drug development, it is important to detect epitope differences in the interaction of the target antigen with Ab^19^. Specifically, epitope binning assay is required to avoid patent disputes and has been particularly relevant in recent long-term patent infringement litigation involving companies such as Sanofi and Amgen^25^. This highlights the importance of the two sdAbs identified in this study with epitope differences to the Repatha. This is attributed to the fact that the domains of sdAbs are often able to target different epitopes in comparison to those of Abs^26,27^.

Notably, in the epitope binning assay (Fig. 2), the primary epitope of PCSK9 must be fully saturated by the primary Ab (captured by the Protein A chip) before the secondary Ab is added. Otherwise, it can lead to false-positive results, erroneously suggesting that two antibodies bind different epitopes of PCSK9. Additionally, it is necessary to optimize the level of the primary Ab captured by the Protein A chip, keeping it below 500 RU. Excessive primary antibody levels may make it challenging to saturate the primary epitope fully, resulting in wastage. Moreover, when adding the secondary Ab, it is necessary to select a primary Ab (captured Ab) with a distinct label (e.g., -his or -strep) if using the primary antibody as a negative control. Otherwise, it can also cause false positives.

For the development of therapeutic anti-PCSK9 Abs, in addition to the epitope binning assay (Fig. 2) and LDL-uptake assay (Fig. 5), WB analyses assays (Fig. 6) of LDLR recovery are also necessary. The recovery of mature LDLR (glycosylated, ~ 150 kDa) serves as an additional evaluation parameter for assessing the inhibition of PCSK9 and promotion of LDL-c metabolism by the Abs. Notably, the immature LDLR (approximately 120 kDa) proteins were weakly detected in immunoblotting experiments, and thus their bands were cut out and not shown in Fig. 6.

Furthermore, in addition to biparatopic Abs, the incorporation of natural trimeric motifs such as human type III procollagen or foldon (Fd) protein can facilitate the design of tri-specific VHH Ab^28,29^. This approach has the potentials to further improve the pharmaceutical effect of the Ab drugs, which represents a direction for future research^30,31^.

Owing to their distinct epitope binding properties, these antibodies (the Repatha and VHH-B11/H2) can contribute to the immunological assay in vitro and the design of immunological kits based on “sandwich” ELISA. By selecting any two Abs, with one acting as the coating Ab and the other as the detecting Ab, an “Ab pair” can be formed in immunological detection assays. This approach can aid in monitoring PCSK9 levels in serum, although the specific epitope of the VHH-H2 binding PCSK9 is not yet well understood. It needs to be explored in future.

Last but not the least, since these B11/H2/B11-H2-Fc Abs have different PCSK9-binding sites with the Repatha, they may act either through an allosteric effect on PCSK9 conformation or by causing PCSK9 multimerization. These findings indicate that some unknown mechanisms induce the PCSK9 inhibition. Further, due to the lack of human PCSK9 transgenic animals and ethical considerations, we did not perform pharmacodynamic tests on animal models, which may be a limitation of this study. However, PCSK9 transgenic animal model construction requires substantial funding, advanced biotechnology, and significant time investment, posing a high barrier for general research institutions^32,33^.

In conclusion, we first designed a novel biparatopic anti-PCSK9 HcAb and explored its lipid-lowering therapeutic potentials in the HepG2 cells model.

## Materials and methods

### Phage display and sdAb screen

A new sdAb selection was performed based on the previously reported phage library^20^. After specific screening, the most positive clones were subjected to Sanger sequencing for further characterization. In the phage-ELSIA assay, three wells of PBS (A1–A3) were used as blank control, and three wells of no-coating PCSK9 antigen (H10–H12) were used as the negative control. The positive clones were recognized by the ratio of OD450 values between the experimental group and the blank group ≥ 2.1. Then the recombinant sdAbs were expressed in the HB2151 *E. coli* system and isolated using Ni-chelating affinity purification.

Subsequently, affinity measurement was performed for the newly identified sdAbs. Briefly, human PCSK9 antigen (Cat# 29698-H08H1, Sinobiological, China) was diluted to 20 μg/mL and coated to a level of approximately 600 RU on the CM5 chip (Cat# 29104988, GE Healthcare, USA). Two-fold series dilutions of sdAbs were injected and flowed through the chip for affinity determination. The reaction temperature was set to 25 °C. The regeneration solution comprised 100 mM glycine solution at pH 2.0, whereas the system solution comprised 1 × PBST. The final affinity results were based on fitted curves generated by the self-built evaluation software of the Biacore T200 (GE Healthcare, USA). Based on these curves, koff and kon values were derived using the equation KD (nM) = koff (1/s)/kon (1/Ms). The Rmax value represented the maximum response value, typically ranging between 0 and 100 RU.

### Epitope binning by SPR

Epitope binning assays were performed using the SPR technology at 25 °C. Firstly, evolocumab (also known as the Repatha, CAS# 1256937-27-5, Amgen, USA), diluted to 2 µg/mL, was captured on the flow cell 2 (FC2) of the Protein A chip (Cat# 29127555, GE Healthcare, USA) at approximately 300 RU level, while FC1 served as the blank control. Subsequently, the PCSK9 protein (his tagged, diluted to 5 µg/mL) was injected into the dual FCs for 90 s. This resulted in the occupancy of all the specific epitopes on PCSK9 by Repatha itself, leading to a plateau in the binding curve. Then, the sdAbs (his-tagged, 5 µg/mL, in PBS) were injected into the dual flow cells for 60 s. If the sdAbs possessed different PCSK9-binding epitopes compared to Repatha, their curves would continue to rise. Conversely, if the sdAbs shared the same epitopes, their curve would remain at a stable RU level. The dissociation time was set to 120 s, and pH 2.0 glycine was taken as the regeneration buffer.

Secondly, VHH-B11-Fc was also captured on flow cell 2 (FC2) of the Protein A chip at approximately 300RU level, with FC1 as the blank control. Next, the PCSK9 protein (his tagged, diluted to 5 µg/mL) was injected into the dual FCs for 90 s. VHH-B11-Fc occupied all its specific epitopes on the hPCSK9, and the hPCSK9’s binding curve would plateau. Subsequently, the sdAbs (his-tagged, 5 µg/mL in PBS) were injected into the dual flow cells for 60 s. The his-tagged VHH-B11 served as the negative control. If the other sdAbs possessed distinct PCSK9-binding epitopes compared to VHH-B11, the curve would continue to rise. Conversely, if the sdAbs shared the same epitopes, the curve would remain at a stable RU level. The dissociation time was also set to 120 s, and the regeneration buffer used was the same as that aforementioned.

### Design and construction of the biparatopic antibody and avidity test

To enhance the avidity and recognition specificity to human PCSK9, the screened sdAbs were tandemly linked with a (G_4_S)_3_ linker and fused to human Fc, forming a novel biparatopic Ab (VHH-VHH-Fc), which was expressed using *pFUSE-hIgG1* vector (Invivogen, USA) in human embryonic kidney 293F cells (HEK293F) cells as described previously (Sinobiological, China)^19^. This is because (G_4_S)_3_ is the very commonly used flexible linker peptide^26,34^. Subsequently, the novel Ab was purified using a Protein A column. Avidity determination of the purified Ab was performed using the SPR technique based on a direct coupling method. Initially, the PCSK9 antigen (his-tagged, 20 µg/mL) was coupled to both FC1 and FC2 of a CM5 chip to a level of approximately 790 RU, followed by the injection (flow-through) of six two-fold dilutions of the Ab (3.125 nM–6.25 nM–12.5 nM–25 nM–50 nM–100 nM). The reaction temperature of the Biacore T200 was also set to 25 °C. For both protocols, a 100 mM glycine solution at pH 2.0 was used as the regeneration solution, and the system solution employed was 1 × PBST. The final avidity results were based on the fitted curves generated by the evaluation software of the Biacore T200. Based on these curves, koff and kon values were derived using the equation KD (nM) = koff (1/s)/kon (1/Ms). The Rmax value represents the maximum response value.

### Eenzyme linked immunosorbent assay (ELISA)

The ELISA was performed to further verify the efficiency of the novel Ab in inhibiting PCSK9’s binding to LDLR. Briefly, LDLR-his protein (Cat# 10231-H08H, Sinobiological, China) was first diluted to a concentration of 1 μg/mL. Subsequently, 100 ng (100 μL) LDLR-his was added to the wells of the ELISA plate and incubated overnight at 4 °C for coating, while no-coating wells were included as blank controls. After three washes with 1 × PBST, the ELISA plate was blocked with 3% BSA at room temperature for 2 h. Simultaneously, the triple series dilutions of the Repatha, VHH-B11-Fc, VHH-H2-Fc, or B11-H2-Fc Abs (200 nM–66.67 nM–22.2 nM–7.4 nM– . . . –0.0102 nM–0.0034 nM) were respectively incubated with 200 ng of PCSK9-hFc per well for 1 h at room temperature. Additionally, 1 × PBST served as the blank control. The incubated antigen-Ab mixture was then added to the ELISA wells as “primary Ab”.

The plate was further incubated for 2 h at room temperature. After five times washes with 1 × PBST, 100 μL of horseradish peroxidase (HRP)-labeled rabbit anti-human IgG secondary Ab, diluted at 1:10,000, was added to each well and incubated for another 1 h at room temperature.

After washing, 100 μL of TMB substrate was added to each well and incubated for 5–10 min in the dark. The chromogenic reaction was terminated by adding 50 μL of 2M sulfuric acid to each well. The plate was placed on the microplate reader and read at OD450nm. The inhibition curve of the Ab-ELSIA assay for PCSK9 binding to LDLR was plotted using the Ab concentration as the horizontal coordinate and the OD450nm value as the vertical coordinate. The PCSK9-inhibiting rates of the tested Abs were calculated as follows: Inhibiting rate = (1 − OD450_Ab_/OD450_PBST_) * 100%.

### LDL-uptake assay in HepG2 cells

HepG2 cells were preserved in our laboratory and seeded in the logarithmic growth phase at a density of 2 × 10^4^ cells/well in 96-well plates (Cat# 423501, Biolegend, USA) in a DMEM medium (Cat# 11965084, Gibco, USA) supplemented with 10% fetal bovine serum (Cat#10099141, Gibco, USA). After 24 h incubation at 37 °C, the cells were divided into six groups. The first group served as the blank control (BC, no addition of PCSK9 and its inhibitor). The second group was served as the negative control (NC, addition of 0.1 μM PCSK9 alone). The remaining four groups consisted of pre-incubated mixtures of 0.1 µM PCSK9 and the respective concentrations of Abs (0.375 μM, 0.750 μM and 1.500 μM) at 37 °C and were added to the cells in triplicate.

Following 1 h incubation at 37 °C, 10 μL of LDL-BODIPY (Cat# L3483, Invitrogen, USA) was added to the cell medium and further incubated for 3 h. After three times washes in Dulbecco’s phosphate buffered saline (Cat# C14190500BT, Gibco, USA), plates were scanned on a Tecan Infinite M1000 PRO (Switzerland). MFI was used to quantify the LDL-uptake level. The following formula was used: PCSK9-inhibiting rate (%) = MFI [(BC group) − (Ab group)]/MFI [(BC group − (NC group)] ∗ 100%.

### Western blotting analyses

To assess the LDLR recovery in the hepatocytes, WB analyses were performed. The grouping method is the same as that in the LDL-uptake. α-tubulin (~ 55 kDa) was served as the internal control. The target protein LDLR and α-tubulin are incubated with different Abs. To avoid non-specific bands, we did not perform co-incubation. Under the same conditions, each sample from six experimental groups was divided equally into two equal parts. And they were applied onto two same 4–20% gradient SDS-PAGE gels, one for the test of LDLR and the other for α-tubulin. Subsequently, the bands on the gels were electro-transferred onto the polyvinylidene difluoride membranes (Cat# ISEQ00010, Millipore, USA) at 400 mA for 2.5 h. The two membranes were incubated with 5% skim milk in 1 × PBST for 1 h at 37 °C to prevent nonspecific binding. Subsequently, one membrane was incubated with a 1: 2 000 dilution of mouse anti-LDLR Ab (Cat# 10231-MM05, Sinobiological, China) at 37 °C with gentle agitation for 1 h. After four washes with 1 × PBST, this membrane was then incubated in HRP-conjugated goat anti-mouse IgG (1: 1000 dilution , Cat# SE131, Solarbio, China) at room temperature for 0.5 h. The other membrane was incubated with the HRP-conjugated rabbit anti-α-tubulin (1: 5000 dilution, Cat# ab185067, Abcam, UK) Ab at room temperature for 0.5 h. Protein detection was performed using an electrochemical luminescence western blot detection system (Cat# P0018FS, Biouniquer, China) in accordance with the manufacturer’s instructions.

### Statistical analyses

All the statistical analyses were performed using the GraphPad PRISM 9 software. The data were expressed as mean ± SD. Statistical significance was determined using Student’s paired* t*-test. A *P* value less than 0.05 was considered statistically significant (*P < 0.05, **P < 0.005, ***P < 0.0005; ns: not significant).

### Ethics approval and consent to participate

This study did not involve human or animal samples and therefore did not require ethical approval. We confirm that all experiments were conducted in accordance with relevant guidelines and regulations.

### Supplementary Information

Supplementary Information.

## Data Availability

All data generated or analysed during this study are included in this published article [and its supplementary information files].

## Abbreviations

(G_4_S)_3_-linker: GlyGlyGlyGlySerGlyGlyGlyGlySerGlyGlyGlyGlySer
Antibody: Ab
BSA: Bovine serum albumin
*Escherichia coli*: *E. coli*
ELISA: Enzyme linked immunosorbent assay
Fc: The constant region fragment of the human immunoglobulin gamma
HcAb: Heavy chain antibody
HEK293F: Human embryonic kidney 293F cells
HepG2: Human hepatoma cells G2
hPCSK9: Human proprotein convertase subtilisin/kexin type 9
HRP: Horseradish peroxidase
VHH-B11: A single domain antibody named by B11; the same is true for VHH-D8/H2
VHH-B11-Fc: A heavy chain antibody named by VHH-B11-Fc
IgG: Immunoglobulin gamma
IPTG: Isopropyl-β-d-thiogalactoside
kDa: Kilo dalton
LDL: Low density lipoprotein
LDL-c: Low density lipoprotein cholesterol
LDLR: Low density lipoprotein receptor
MFI: Mean fluorescence intensity
OD450: The optical density value at 450 nm
PCSK9: Proprotein convertase subtilisin/kexin type 9
RU: Response units
sdAb: Single domain antibody
SPR: Surface plasmon resonance
VHH: High variable region of the heavy chain antibody
PBST: Phosphate buffer solution with 0.05% tween 20
