# Supplementary material for: Biparatopic anti-PCSK9 antibody enhances the LDL-uptake in HepG2 cells
Source: Sci Rep. 2024 Jul 3;14:15331. doi: 10.1038/s41598-024-66290-9 (PMC11222478; doi:10.1038/s41598-024-66290-9)
Supplement: Supplementary file 1 — Supplementary Information. [file 41598_2024_66290_MOESM1_ESM.pdf]

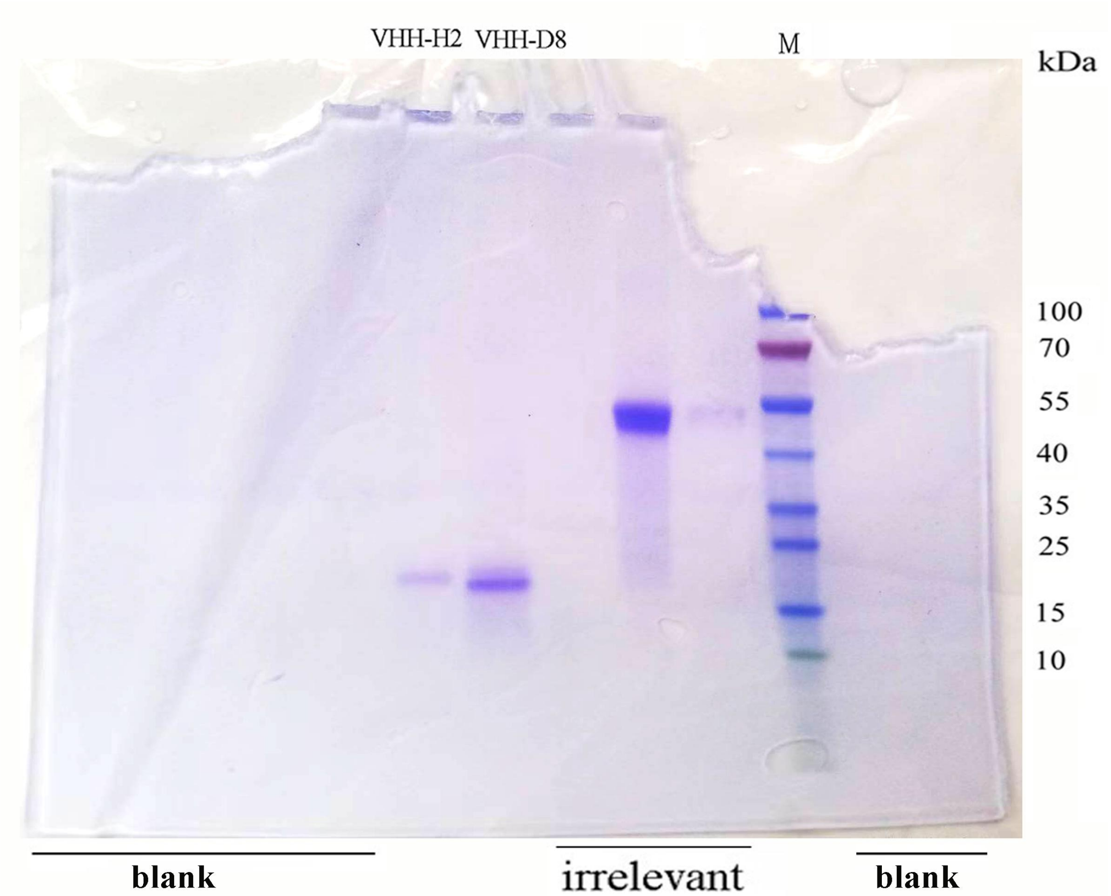
VHH-H2
VHH-D8
M
kDa
100
70
55
40
35
25
15
10
blank
irrelevant
blank

Fig. S1

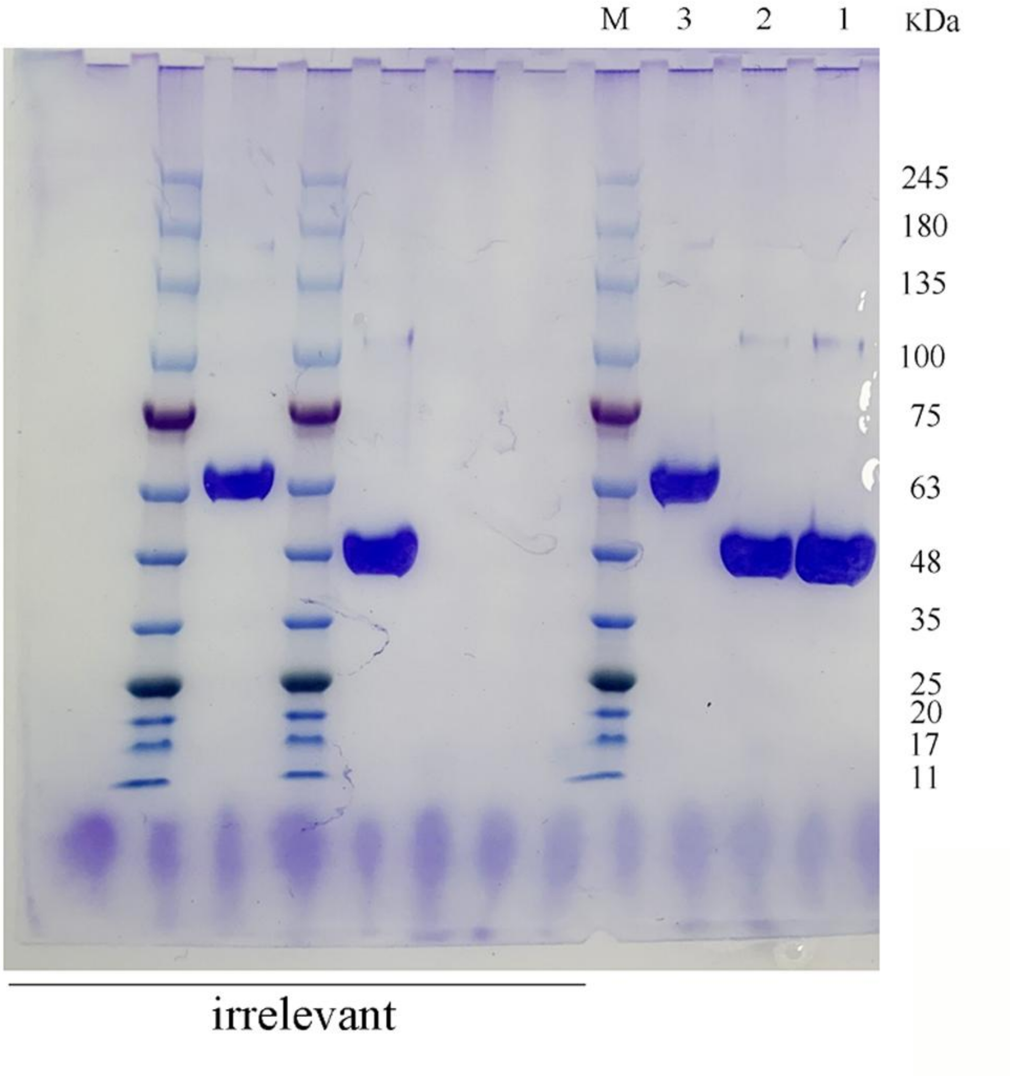

Fig. S2

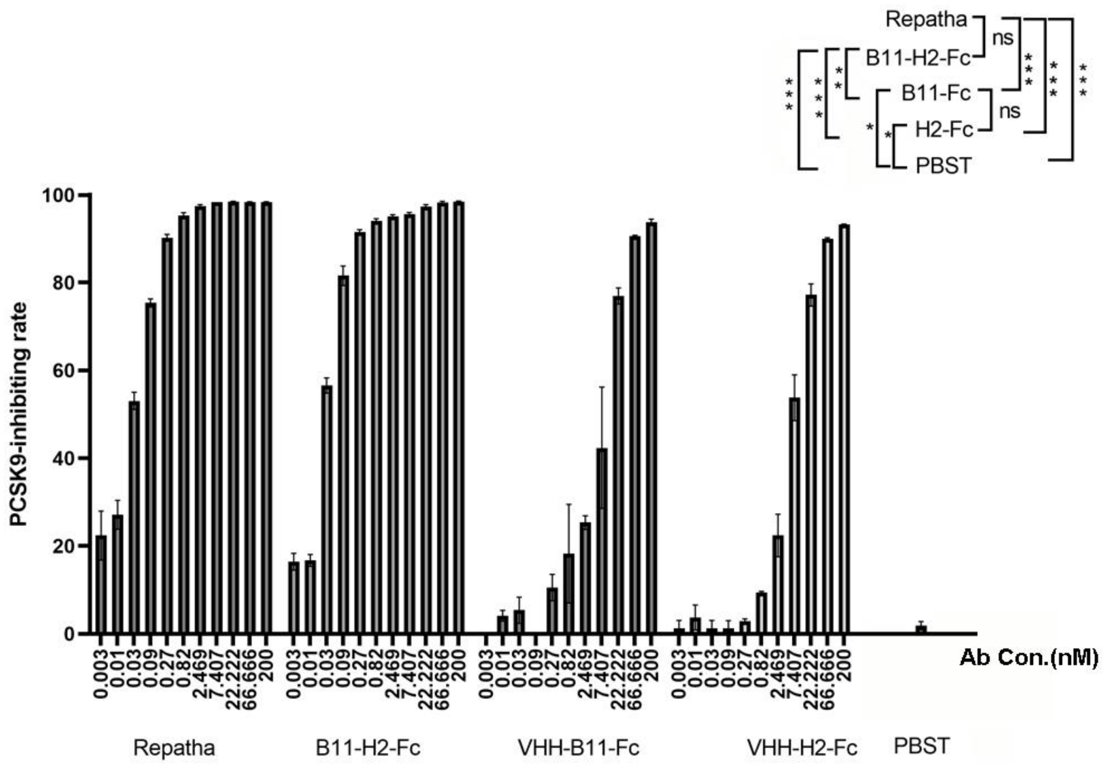
Repatha
B11-H2-Fc
B11-Fc
H2-Fc
PBST
ns
ns
***
**
*
**
***
***
***
PCSK9-inhibiting rate
100
80
60
40
20
0
Ab Con.(nM)
0.003
0.01
0.03
0.09
0.27
0.82
2.469
7.407
22.222
66.666
200
Repatha
B11-H2-Fc
VHH-B11-Fc
VHH-H2-Fc
PBST

Fig. S3

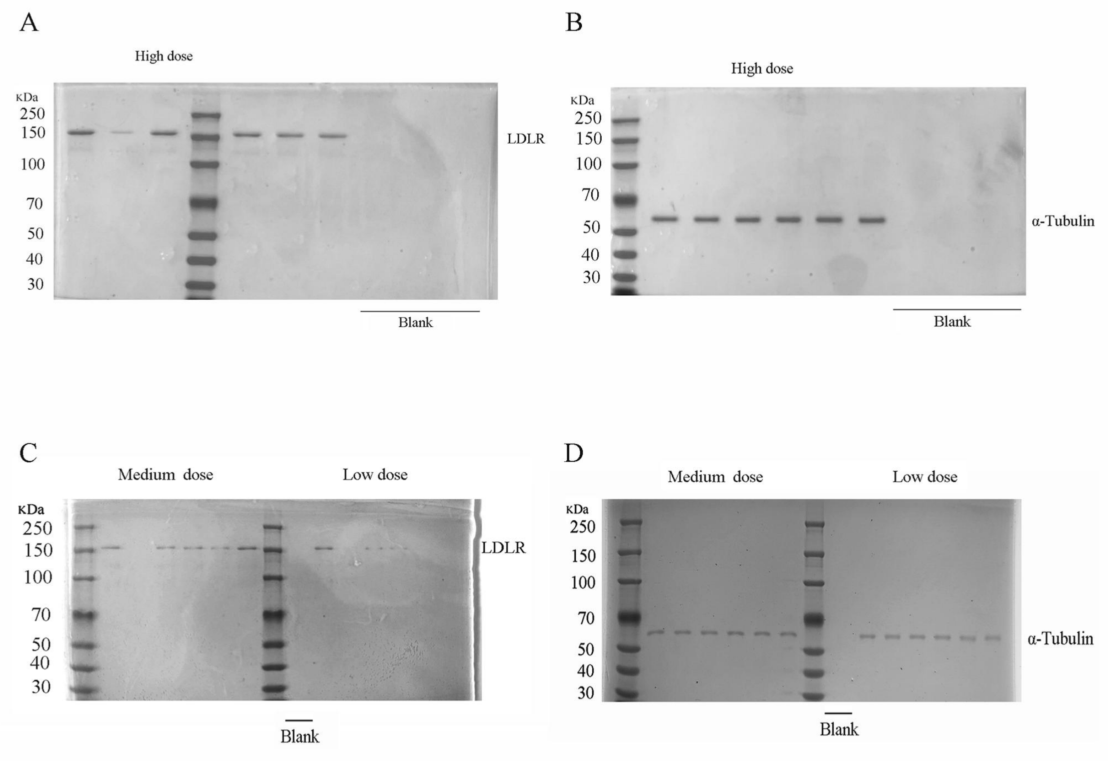
A
High dose
KDa
250
150
100
70
50
40
30
LDLR
Blank
B
High dose
KDa
250
150
100
70
50
40
30
α-Tubulin
Blank
C
Medium dose
Low dose
KDa
250
150
100
70
50
40
30
LDLR
Blank
D
Medium dose
Low dose
KDa
250
150
100
70
50
40
30
α-Tubulin
Blank

Fig. S4

Table S1

SdAb ID	Amino acid sequences
VHH-D8	QVQLQESGGGLVQAGGSLRVSCVASGSTFSGYAMAWF RQAPGKEREFVAAIEREIPGHPAWSGLTYYADSKKGRFT ISRDNAKNTVYLQMNSLKSEDTAVYYCAAGLKYPAQK HYDYDYWGQGTQVTVPA
VHH-H2	QVQLQESGGGLVQAGGSLRLSCAASGRTFSDYAVGWF RQAPGKEREFVAGIGWSGGQTTYADSVKGRFTISRDNA KDTVYLQMNSLKPEDTAVYVCAASFLVIPGTVKTRYDS WGQGTQVTVPA
VHH-B11	EVQLVESGGGSVQAGGSLRLSCTVSGYTYSSNCMGWF RQAPGKEHEGVASIYIGGGSTYYADSVKGRFTISQDNA KNTVYLQMNSLKPEDTAMYYCAVGCQGLVDFGYWDQ GTQVTVSS

Figure S1 The original SDS-PAGE gel photo of the Fig. 1B

Figure S2 The original SDS-PAGE gel photo of the Fig. 3C

Figure S3 The bar chart of the inhibiting rate of the ELISA assay

The horizontal coordinate represents the different Ab groups on the different concentrations. The vertical axis represents the PCSK9-inhibitting rate of the ELISA assay. The data were expressed as mean ±SD. Statistical significance was determined using Student's paired *t*-test. P<0.05 was considered as statistically significant, compared with the negative control group (*P<0.05, **P<0.005, ***P<0.0005, ns: not significant).

Figure S4 The original western blotting photos of the Fig. 6

The original western blotting photos of the Fig. 6. The western blotting and quantitative analyses were performed to determine the expression level of LDLR (~150kDa) of hepatocytes. The results are presented in the form of high (1.5 μM) (**A**), medium (0.75 μM) and low (0.375 μM) (**C**) doses. The α-Tubulin (~55kDa) was served as the internal control protein (B & D). "Blank" refers to that there are no samples in these lanes.

Table S1 The amino acid sequences of the sdAbs

Note: sdAb ID represents the name of the single domain antibody.
